# 

**Published:** 1872-01

**Authors:** 


All communications and exchanges should be addressed to the Atlanta Medical and Surgical Journal, care of "The Plantation Publishing Company," Atlanta, Georgia.

